# Targeting melanoma residual disease by USP7

**DOI:** 10.18632/oncotarget.26497

**Published:** 2018-12-25

**Authors:** Monika Vishnoi, Dario Marchetti

**Affiliations:** Dario Marchetti: Biomarker Research Program Center, Houston Methodist Research Institute, Houston, TX, USA

**Keywords:** USP7, bone marrow resident cells, circulating tumor cells, melanoma, liquid biopsy

Melanoma metastasis is an aggressive disease which exhibit a latency period between the onset of primary and metastatic tumors. Overall patient survival is reduced from 98% to 19%, respectively [1]. While pre-existing adjuvant and neo-adjuvant immunotherapies improve clinical outcome, they are inefficient to provide cure in metastatic melanoma settings [2]. Accordingly, there is the urgent need to develop novel therapeutic regimens which can prevent the colonization of tumor cells at distant sites [3]. Non-invasive liquid biopsy procedures represent an effective approach to monitor clinical progression in real-time and to identify candidate biomarkers in early *vs* late disease stages [4, 5]. They will provide precision oncology therapy to inhibit disease recurrence, ultimately reducing cancer mortality.

During metastasis latency period, disseminated circulating tumor cells (CTCs), known “seeds” of metastasis, home to and reside at distant organs, undergoing temporal-spatial selection to acquire metastatic traits which lead to disease recurrence [2, 3]. In the clinically asymptomatic phase, CTCs follow a stochastic route to reside for prolonged periods in bone marrow (BM), a reservoir of CTCs. These BM-resident CTCs (BMRTCs) interact with the surrounding environment to maintain balance between stem cell renewal and metastatic abilities required for their survival and homing [4, 5]. Targeting BMRTC stem-cell properties to eliminate residual cells or delaying their ability to acquire metastatic traits can therefore be a promising therapeutic strategy to delay or overcome metastasis [3].

We have provided first-time evidence recapitulating the biology of clinically undetectable melanoma metastasis by injection of Lin-neg/CTC-enriched population (DAPI-/CD45-/CD34-/CD73-/CD90-/CD105- cells) sorted from blood of melanoma patients in CTC-derived xenografts (CDXs) [6]. We discovered high heterogeneity in these “biomarker-agnostic” CTCs which also beared distinct transcriptional signatures of BMRTCs *vs* CTCs isolated in parallel from CDXs. Further, we assessed the prognostic relevance of low-proliferating, however metastasis-competent BMRTCs, and identified protein ubiquitination as the most significantly altered canonical pathway (BMRTCs *vs* CTCs) [6]. The ubiquitin proteasome system is a complex network of proteins responsible for intracellular protein degradation and turnover. Recent advances have resulted in the development of a new generation of deubiquitinating enzymes and proteasome inhibitors targeting previously “undruggable” ubiquitin proteasome targets (e.g., tumor suppressors), along with abilities for disrupting protein-protein interactions in ligases overseeing their degradation [7, 8]. We evaluated USP7/HAUSP, a deubiquitinase which we found associated with worst overall survival of melanoma patients. USP7 is involved in multiple functions of cell maintenance by regulating post-translational modifications of key tumor suppressors such as p53 and PTEN [9, 10]. Pharmacological inhibition of USP7 in melanoma CDXs eliminated minimal residual disease at metastatic sites and arrested BMRTCs at BM locales [6]. USP7 expression correlated with PTEN expression, and inhibition of USP7 restored nuclear localization of PTEN which is essential for normal hematopoietic stem-cell functions. PTEN plays critical roles switching hematopoietic stem cells quiescence to a metastatic state by the regulation of G1 to S cell-cycle transition. USP7 inhibition arrested BMRTCs residence at BM, conceivably mediating G1/S phase of cell-cycle progression. Targeting residual melanoma cells by USP7 inhibition induced p53/p21-mediated senescent phenotype which may lead to activation of apoptotic pathways [6, 10]. We demonstrated that USP7 modulated the senescent phenotype of BMRTCs while reducing melanoma micro-metastasis. This suggests that different microenvironment cues regulate USP7 in melanoma cells and will determine the fate and function of BMRTCs. USP7 maintains regulatory T-cell (Treg) function by deubiquitination of transcription factors FOXP3, Notch1, etc. [10]. USP7-mediated direct or indirect NF-κB deubiquitination asserts its role in inflammation and immune signaling pathways to regulate various cellular functions such as differentiation, survival and proliferation [10](see also Figure 1). Interactions between USP7 and immune cells might facilitate the stem-cell phenotype of BMRTCs and provide a pre-metastatic niche for effective organ colonization. Further USP7 mechanistic insights will provide effective strategies to target residual cells. For example, USP7 may regulate the extrinsic and intrinsic behavior of BMRTCs, either by acquiring genetic traits (e.g., APC/SMAD acquired mutations) during disease progression or by interacting with the surrounding microenvironment. Cross-talks of tumor cells with osteoblasts, myeloid cells, natural-killer cells, T-cells, etc., may discriminate the fate of disseminated cells to remain into a quiescent state or switch to a fully competent metastatic cell. Transcriptional profiling revealed that USP7 affected multiple BMRTC functions such as migration, invasion, and reseeding in the circulation, possibly altering cell growth and proliferative capacities during the metastatic cascade [6].

**Figure 1:**
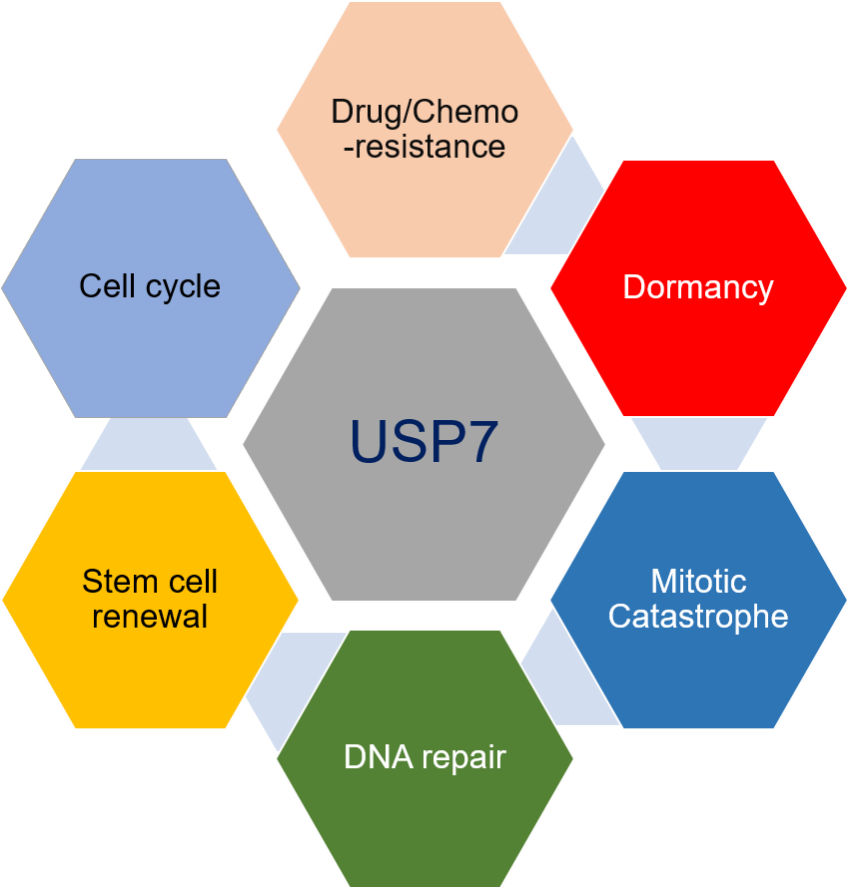
Roles of USP7 regulating CTC properties

USP7 is an ideal anticancer target molecule which modulate BMRTCs at several metastatic steps [6]. Therefore, its pro-oncogenic and immune-modulatory roles to regulate survival, metastatic initiation and colonization of residual cell population during cancer asymptomatic phase warrants further investigation (Figure 1).

Concluding, liquid biopsy-based screening is poised to provide new surrogate biomarkers to reduce the risk of metastatic recurrence. Pre-clinical studies to assess the therapeutic relevance of potent, substrate-specific, druggable, small-molecule USP7 inhibitors in large patient cohort need to be considered as a valuable option [7, 8]. Additional investigations on USP7 roles in cancer progression employing more selective/drug-like inhibitors than ones used in our study will have important implications to develop precise therapy. Potential synergistic combinations of standard cancer therapeutic drugs with USP7 inhibitors in clinical settings will ultimately impact cancer patient survival in general, melanoma in particular.

## References

[1] Howlader N SEER cancer statistics review. 1975-2014.

[2] Luke JJ (2017). Nat Rev Clin Oncol.

[3] Anderson RL (2018 Dec 4). Nat Rev Clin Oncol.

[4] Müller V (2010). Eur J Cancer.

[5] Joose SA (2016). Cancer Metastasis Rev.

[6] Vishnoi M (2018). Cancer Res.

[7] Turnbull AP (2017). Nature.

[8] Desroses M (2017). Cell Chemical Biology.

[9] Zhou J (2018). Med. Chem.

[10] Wu J (2018). J Med. Chem.

